# Patients’ preferences in selecting family physician in primary health centers: a qualitative-quantitative approach

**DOI:** 10.1186/s12875-020-01181-2

**Published:** 2020-06-11

**Authors:** Farnaz Khatami, Mohammad Shariati, Leila Khedmat, Maryam Bahmani

**Affiliations:** 1 Department of Community Medicine, Tehran University of Medical Sciences, Tehran, Iran; 2 Department of Family Medicine, Ziaeian Hospital, Tehran University of Medical Sciences, Tehran, Iran; 3 Health Management Research Center, Baqiyatallah University of Medical Sciences, Tehran, Iran

**Keywords:** Family physician, Patient satisfaction, Primary care, Health seeking behavior, Continuity of care

## Abstract

**Background:**

The role of family physicians (FPs) in the metropolitan area is critical in identifying risk factors for disease prevention/control and health promotion in various age groups. Understanding patients’ preferences and interests in choosing a FP can be an effective and fundamental step in the success of this program. In this study factors affecting the FP selection by Iranian patients referred to health centers in the most populous areas in the south of Tehran were assessed and ranked.

**Methods:**

A sequential mixed-method (qualitative-quantitative) triangulation approach was designed with three subject groups of patients, physicians, and health officials. The Framework method was used to analyze interviews transcribed verbatim. After implementing an iterative thematic process, a 26-item quantitative questionnaire with high validity and reliability was drafted to evaluate the different factors. A convenient sampling method was used to select 400 subjects on a population-based scale to quantitatively rank the most critical selection factors as a mean score of items.

**Results:**

The selection factors were divided into six centralized codes, including FPs’ ethics, individual, professional and performance factors; patients’ underlying disease and individual health, and disease-related factors, office’s location and management factors, democracy factors, economic factors, and social factors. After filling out the questionnaires, the most important factors in selecting FP were a specialist degree in family medicine (FM) (4.49 ± 0.70), performing accurate examinations with receiving a detailed medical history (4.43 ± 0.68), and spending enough time to visit patients (4.28 ± 0.75), respectively. However, the parameters such as being a fellow-citizen, being the same gender, and physician’s appearance were of the least importance.

**Conclusion:**

There is a possibility to screen the most important factors affecting the FP choice through the combination of qualitative and quantitative studies. The first and last patients’ priority was physicians’ specialty in FM and being a fellow-citizen with them, respectively. The clinical and administrative healthcare systems should schedule the entire implementation process to oversee the doctor’s professional commitment and setting the visit times of FP.

## Background

Health is the foundation of the social, political, and cultural development of all human societies. As it is of particular importance in the formation of infrastructure in different parts of society, the ultimate goal of any country’s healthcare system is to improve the health of individuals so that they can participate in social and economic activities with adequate health [1]. In this regard, attention has been paid to social inequalities in access to health services and followed by the need for reform aiming at increasing the productivity of the healthcare system has begun in many areas of the world [2]. One of the most successful and comprehensive health plans in the developing and developed countries is presenting the package of health care services recommended by family physicians (FPs). The FP is responsible for providing health services within a defined range, without any prejudice to age, sex, and socioeconomic status to the individuals, families, and communities [3–5]. In Iran, the FP program was launched in rural areas and cities with fewer than 20,000 populations. It is currently pursuing its output in urban areas in several Iranian provinces [6]. In Iran, Essential Package of Health Services (EPHS) in primary care is related to services provided by a team in the health system in the rural and urban areas, which is usually located close to patients’ residences. The EPHS in primary care in Iran includes: (i) prevention: immunization, prevention, and control of communicable and non-communicable diseases, prevention of unwanted pregnancies, oral hygiene, and mental health, (ii) health promotion: health education, and learning healthy lifestyles and life skills, (iii) early treatment and emergency management: visiting the office, diagnosing and treating diseases, performing simple surgeries such as stitches, vasectomy or circumcision, injections, dressing, home visits, and cardio-respiratory resuscitation, (iv) referral: eligible patients for secondary or third level specialty care, and (v) health management: the record of population’s health information, advocacy, and monitoring the work of health team members [3, 7].

Therefore, the presence of family physicians is a necessity to meet the public health needs, address the unnecessary increase in health costs, and prevent its adverse impact on public health [8, 9]. On the other hand, one of the essential principles for the provision and development of health care services is the need to pay attention to the collaboration and participation of patients and physicians in the program. The active involvement of all patients is also essential for its successful implementation and requires effective communication with patients, families, and the community to achieve an integrated and efficient model [10].

Different factors affect the patients’ thoughts to choose a primary care doctor. In recent years, the issue of doctor selection, especially in developed countries, has received serious attention from health policymakers and health insurance organizations [7]. Patient-led physician selection leads to greater competitiveness of physicians, improved service quality, better access to medical services, and increased care efficiency [3, 11]. However, there is a minimal research background on determinant factors in choosing the FP by patients in Iranian populations. Despite the generalization of the FP program in Iranian urban society, a comprehensive study to determine the most critical personal-social factors in the selection of FPs by patients has not been conducted yet. Therefore, the current study aimed to evaluate factors affecting FP selection in the covered population of health centers in the south of Tehran.

## Methods

### Study design and participants

A qualitative-quantitative mixed study was carried out between 2018 and 2019. Two approaches of relevant literature review and interview process were applied to assess the influencing factors on the FP selection. The selected strategy of interviewees was purposive that included three groups of physicians, health system managers (HSMs), and patients referring to the healthcare centers, who were selected with a triangulation method. This technique is typically used to evaluate an issue by explaining its different aspects. The triangulation method ensures data validation via cross verification from more than two sources [12]. According to the obtained results in this studying phase, the questionnaire was developed based on the internal expert panels’ (IEP) and some participants’ comments and distributed among 400 participants to identify patients’ priorities in choosing an FP. The sample size (*n*) was calculated using the following formula (Eq. 1):

1 $$ n=\raisebox{1ex}{${z}^2p\left(1-p\right)$}\!\left/ \!\raisebox{-1ex}{${d}^2$}\right. $$

where *z*, *p*, and *d* are the 95% confidence level based on the standard normal distribution (z = 1.96), the estimated proportion of the population presenting the characteristic (assuming at least 50% in the selected priority, *p* = 0.5), and tolerated margin of error (*d* = 5%), respectively [13]. Accordingly, the sample size for the current study is ~ 385. However, based on the attrition risk of 10%, the sample size to compensate was reached to 400 subjects.

Then, the designed questionnaire was distributed to a population of patients referring to hospitals and health centers in the south of Tehran through available sampling in a field study. The voluntary individuals with at least 18 years old were included in this study. Illiterate patients were asked to assist the questioner or patient companion by reading the questions.

### Survey development and administration

#### Literature search and screening

A review of the literature was carried out to extract the most important factors influencing FP selection in books, articles, and texts (Table 1). The main keywords used to search in different scientific databases within 1987–2018 were: family physician, patient satisfaction, primary care, health care behavior, and continuity of care. About 80 articles, including studies conducted in Iran and other countries, were reviewed, and finally, 57 more relevant articles were selected as the reference.

**Table 1:** The most critical factors affecting selection in the literature review (familiarization stage)

Factor type	Criteria
Individual	Doctor’s/Patient’s age, gender, and ethnicity, Patient’s education level, Patient confidence in physician performance, High communication skills with patients, The effectiveness of the physician’s actions on the previous visit (Patients and their family members), and Type and severity of the disease
Behavioral-ethical	Punctuality, Confident and trustworthy, Responsibility, Doctor’s sympathy, and Answering to patients’ questions with patience
Professional and performance	Having high scientific level, Doctor’s skill and expertise, Assigning sufficient time to visit patient, High carefulness in medical examination, Introducing and referring by other colleagues or specialists, Visiting patients privately, not in the presence of others, Providing the complete health services package, Presenting information on diagnostic, therapeutic and caring measures and providing appropriate solutions, and Avoiding any unnecessary referral
Locational, environmental and management	Proximity of doctor’s office, Easy and convenient access to the doctor’s office, Well-equipped and stylish workplace, Doctor’s cleanness, Comfortable and large waiting room, Short waiting time, Scheduled visits without any delay, Using efficient assistants in healthcare centers, and Media and advertising (women were more influenced)
Economic	Diversity and low-cost of complementary health services, Doctor’s visit price, And acceptance of health insurance
Sociocultural	Recommended by friends and acquaintances, Publicity, Patients’ awareness level, and Treatment level based on the regional socioeconomic status

#### Research team and reflexivity

Four qualitative researchers involved with the research process. All were medical doctors. One was male and three were female. They had experience in designing qualitative studies, writing qualitative books, and teaching in this field. To reduce bias, the team had not close familiarity with participants. Interviewees were selected based on their experiences with triangular techniques. The research team and also the aims of the study were explained to the subjects and entered into the study if they wish to cooperate and contribute with the team to promoting community health.

#### Interview procedure

A step-by-step guide on how to properly conduct a face to face interview was applied, and after transcripts confirmed by participants, the content was analyzed according to the complementary viewpoints of an IEP. Interviews in each part were continued until EQIs were satisfied that the data showed saturation. Three population groups including physicians (*n* = 47), HSMs (*n* = 5), and patients (*n* = 30) were interviewed as follows: The group of physicians consisted of three categories: (i) specialist doctors (SDs), (ii) family medicine residents (FMRs), and (iii) general practitioners (GPs) who were active as FPs in the rural and urban area. The interview open-ended questions of each group were designed by the research team and piloted by the target groups and the necessary corrections were made and finalized. It is worth noting that only two health managers and three physicians due to busy, not cooperate with us for an interview.

#### Interview with physicians’ group

In the first category, FMRs of Tehran University of Medical Sciences (TUMS) were interviewed in a 20-person group meeting. At the beginning of the meeting, the research topic and its purposes and applications were explained, and any ambiguities were resolved. The answers were kept confidential, and the FMRs were then asked to provide their comments on the sheet if they were satisfied with the cooperation. Ongoing verbal interviews were developed with open-ended questions. In the second category, a group of SDs of the hospital from clinical faculty members at TUMS was interviewed in person with open and in-depth questions. All interviews were then recorded and transcribed. In the third category, GPs, who acted as FPs, were interviewed. For comprehensive interviews, urban FPs occupied in the national pilot of the FP plan in Mazandaran province (Amol city, Iran) with at least 7 years of work experience, and rural FPs in healthcare centers of the south of Tehran with 3–10 year work experience were considered. The experienced qualitative interviewers (EQIs) conducted interviews for 20–45 min, audio-recorded, and transcribed verbatim. Field memos documented immediately by the interviewer at the interview time.

#### Interview with health system managers’ group

Open and in-depth interviews with HSMs were conducted after multiple coordination and verbal consent. Each interview with HSMs with a management history of 17–28 years lasted from 40 min to an hour in workplaces.

#### Interview with patients’ group

The subjects were interviewed from referrals to Ziaeian Hospital and healthcare centers in the south of Tehran after the oral and written informed consent. According to the expert instructors’ opinions, 2–3 general questions of the main factors obtained from the literature review as a result of the content analysis were asked. The in-person, individual, semi-structured qualitative interviews for 20–30 min were conducted with patients.

#### Qualitative data analysis

The 5 main stages thematic framework analysis method was used to analyze the qualitative data obtained from the conducted interviews and included: (i) familiarization, (ii) identifying a thematic framework, (iii) indexing, (iv) charting, and (v) mapping and interpretation [12]. The familiarization phase was based on the principle of immersion, by repeated reading of transcripts, and written notes taken during the interview, line by line, by two coders. This step aimed to list key ideas and recurrent themes and to correctly edit a summary of the content of each interview (Table 2). In this table, items and interviewees’ characteristics were displayed in the columns and rows, respectively. After sequential comparisons for each item category, a framework entitled “sub-theme” was selected (Table 3). In the indexing stage, the thematic framework was used in a textual form by explaining the transcripts with the principal and centralized codes from the index. The theme and sub-theme were centralized and principal codes, respectively. The codes were discussed by two coders to make coding decisions. For instance, each interviewee in HSMs group was explained as “M” so that “M1” to “M5” indicates the participant number of 1 to 5. Similarly, relevant codes were considered for the rest of the participating groups. Table 4 was then composed of selected summaries of viewpoints in the ‘charting’ stage. A table was separately tabulated for each group of interviewees by specifying their opinions with an appropriate code in each column, while the principal and centralized codes were defined in the table rows. In the final step of ‘mapping and interpretation’, tables drawn for each interview group were put together and compared to find correlations between themes with a view to providing clarifications for the results [14, 15]. An update was performed in the analysis process of the thematic framework regarding certain coded items that started to cluster, and others separated.

**Table 2:** A list of the main factors affecting the FP selection by patients from the perspective of patients, HSMs, and physicians (familiarization stage)

Groups	Influential factors in patients’ choice
patients (*n* = 30)	Punctual, Careful examination, Being a specialist, Medical experience, Higher age (older), High scientific level, Explain and provide information to the patient, Listen and pay attention to patient talk, Advising the patient about his or her illness, Doctor’s gender, Public or private workplace, Well-equipped and stylish workplace, Proximity of the doctor’s office, The availability to the physician in more weekdays, Satisfaction of other patients, Having previous successful treatment experiences with the selected doctor, Correct diagnosis, Cost-effective examination, And proper behavior.
HSMs (*n* = 5)	Appropriate behavior and interaction with patients, High communication skills with patients, Listen patiently to patient talk, Having a good ethics and personality based on respect, Being kind, regular and punctual, Easy availability to the physician with short waiting time, Easy access to the doctor’s office, The proximity of other health services (e.g., laboratories and pharmacy) to the doctor’s office, Doctors’ office cleaning and sanitary maintenance, Having a neat, clean-cut appearance, Looks like a doctor (by uniform, stethoscope, etc.), Being middle-aged and handsome, Having the common dialect and ethnicity (e.g., Fars, Turk, Kord, Lor, etc.) between doctor and patient, Being fellow-citizen, Being the same gender, Doctor’s reputation, Being holistic (considering the patient’s entire physical-mental condition along with her/his current illness), Being skillful and experienced, Having an excellent scientific level and clinical skills, High carefulness in medical examination and biography, Publicity, Give patients adequate information about diseases and necessary guidance or training, Being accountable to the patient, Keep patients focused during office visits, Follow up and continuity of care, The effectiveness of previous treatment, The patient’s practical experience of physician actions, The trust level of patients in physicians, Cost-effective examination, Satisfaction and experiences of other patients, patients’ religious beliefs, Respecting the traditions and beliefs of patients, patients’ literacy and awareness level, And the disease severity (making the patient more careful and obsessed in choosing a physician)
FMRs (*n* = 20)	Physician knowledge and awareness, The treatment effectiveness, Good ethics (patience, politeness, behavior and communication), Keeping the patient’s medical secrets, Proximity to the doctor’s office, Cost-effective examination, Complementary services at the center to avoid any unnecessary referral of patients for conducting paraclinical or interventional measures, Getting to know the cultural beliefs of the patients of the region, Knowing the level of scientific literacy of the regional patients’ children who are studying and working in another city, Enough awareness of religious beliefs of the regional patients, Awareness of the level of public expectations of the area physician’s performance, Required ability to adequately treat patients and not refer them too much for diagnostic trials, And providing a suitable alternative in times of drug shortage or lack of laboratory facilities
GPs (Rural FPs) (*n* = 6)	Gender (Women often refer to a female doctor), Being a specialist, High medical experience, Having good ethics and personality based on respect, Effective communication and empathy with the patient, Follow up and continuity of care, Other patients’ satisfaction with the treatment outcome, The effectiveness of previous treatment, Presenting the correct diagnosis and rapid treatment, Keeping up to date on scientific information, Give patients adequate information about their diseases, Presenting the necessary training to prevent patient’s illness complications, The referral to the physician who complies with patient requests, Government-funded healthcare centers (with more referrals due to the less payment), And doctors occupied in the private sector (owing to their better care and higher scientific level)
GPs (Urban FPs) (*n* = 6)	Having good behavior and ethics based on respect, Being patient and trusted, Having up-to-date knowledge and high scientific level, Keeping the patient’s medical secrets, Having the effective communication and empathy with the patient, Avoiding unnecessary referral of patients for conducting paraclinical and interventional tests, Correct and necessary referral of patients to other medical centers, Do not be a strict physician, The availability to the physician in most weekdays, High availability to additional services (e.g., sterile injection) in the nearness of physician’s office, Proximity to the doctor’s office, Short waiting time to visit, Regular follow-up and responsibility, Assigning sufficient time to visit, Work experience and reputation, The effectiveness of previous treatment, And satisfaction and experiences of other family members,
SDs (*n* = 15)	Good morals, Doctor’s gender, Punctuality, Honesty, Being holistic, High communication skills with patients and favorable answering to their questions with patience, Proper professional behavior, Avoiding any unnecessary referral, Having up-to-date knowledge and high scientific level, Doctor’s reputation, skill and expertise, Proximity of doctor’s office, Low doctor’s visit price and acceptance of health insurance, Good secretary, Recommended by friends and acquaintances, Geographical location of doctor’s office, Having the required information (office’s phone, and doctor’s mobile), The effectiveness of the physician’s actions on the previous visit(s), Satisfaction and experiences of other patients, Keeping the patient’s medical secrets, Having a neat, clean-cut appearance, High carefulness in medical examination and biography, Assigning sufficient time to visit patient

**Table 3:** Definitions and identifying a thematic framework in the qualitative study

Code no.	Theme	Sub-theme	Code definition
1	F	F1	Ethics factors
		F2	Individual factors
		F3	Professional and performance factor
2	P	P1	Underlying disease and individual health factors
		P2	Disease-related factors
3	C	C1	Location factors
		C2	Management factors
4	D	D	Diplomacy factors
5	E	E	Economic factors
6	S	S	Social factors

*F* Family physician, *P* Patient, *C* Clinic, *D* Diplomacy, *E* Economic, *S* Social

**Table 4:** Charting stage of framework from the viewpoints of HSMs and physicians

**Code**^a^	**HSMs sub-groups**					**Percent (%)**
	M1	M2	M3	M4	M5	
F1-M	■^**b**^	■	■	■	■	100
F2-M		■	■	■	■	80
F3-M	■	■	■	■	■	100
P1-M				■		20
P2-M	■	■	■	■	■	100
C1-M	■	■	■	■		80
C2-M		■	■			40
D-M			■	■		40
E-M	■		■	■		60
S-M	■	■		■	■	80
**Code**^a^	**Physician sub-groups**					**Percent (%)**
	G1 (Rural FPs)		G2 (Urban FPs)	G3 (SDs)	G4 (FMRs)	
F1-G	□		□	□	□	100
F2-G	□		□	□	□	100
F3-G	□		□	□	□	100
P1-G						0
P2-G	□		□	□	□	100
C1-G			□	□	□	75
C2-G			□	□	□	75
D-G						0
E-G	□			□	□	75
S-G	□		□	□	□	100

^a^M and G show the interviewee number in HSMs and physicians, respectively (for example, M1 shows HSM no. 1, G5 reveals physician no. 5 for rural and urban FP, or SD, or FMR)

^b^F1M1 code explains ethics factors mentioned by HSM no. 1

#### Questionnaire design

All the influencing factors by incorporating the themes mentioned by each of the interviewed groups were identified. These fundamental elements emerged with the principal and centralized codes in terms of 43 questions. These question items were then evaluated by the IEP and some participants to present specialized corrections and dedicated processing strategies. After the revision step, frequent questions with a centralized or principal code were put together. Some of the questions were eliminated due to raising unreasonable expectations. According to the experts’ opinions, 26 of the original 43 questions in a short and precise form were arranged. A draft of a questionnaire was then constructed with two main sections of demographic data (e.g., gender, marital status, education level, and health insurance status), and questions related to the various factors affecting the choice of FP. The 26-item questions consisted of four centralized codes and five principal codes. In general, items included questions related to health-related physical factors (6 cases), patient-related factors (2 cases), social factors (5 cases), and physician-related factors (13 cases). Answers to questions for each item were presented on a 5-point Likert scale. Items were rated based on a 5-point Likert scale for levels of satisfaction and importance. Answers ranged from labels of “dissatisfied” and “not very important” (scored as 1.0) to “very satisfied” and “very important” (scored as 5.0); with the total score sum of 26–130.

#### Questionnaire validity and reliability

The qualitative content validity of the developed questionnaire was assessed based on the viewpoints of 20 patients of the studied population and ten individuals of IEP, who had research experience or worked in the field. Twenty participants initially completed the questionnaire to determine whether the designed questions were ambiguous or not? Fortunately, there was not a significant problem in the fluency and understandability of the items so that the response rate ranged from 5 to 85%. Subsequently, 10 IEP members confirmed the re-revised questionnaire by presenting more specific recommendations and better and more obvious questions. In the quantitative content validity, confidence is maintained to select the most important and correct content in a data collection tool. In this assessment, the experts are asked to agree whether an item is essential for operating a construct in a set of items or not? Accordingly, the content validity ratio (CVR) was determined by the following equation (Eq. 2):

2 $$ CVR=\raisebox{1ex}{$\left({N}_e-\frac{N}{2}\right)$}\!\left/ \!\raisebox{-1ex}{$\frac{N}{2}$}\right. $$

where the *N*_e_ and *N* are the number of panelists indicating “essential” and the total number of panelists, respectively [16].

The Lawshe table was used to estimate the numeric value of CVR. The minimum acceptable CVR (MA-CVR) according to the count of scoring panelists. The MA-CVR for each item should be 0.62 as the number of experts was 10 in this study [17]. In this study, the MA-CVR for each item present in the prepared questionnaire was more than 0.62. The Waltz and Bausell’s method was used to assess the content validity index (CVI) for each item by dividing the number of experts who ranked the items as compatible or full compatible for each criterion (relevancy, clarity, and simplicity) to the total number of experts [18]. The mean value of the three criteria was considered as the total CVI for each item. Overall, a CVI value of more than 0.79 for each item was appropriate to be retained [19]. The mean of CVI for the developed questionnaire was calculated to be 0.88. The questionnaire’s reliability was evaluated by calculating the internal consistency reliability coefficient “Cronbach’s alpha”. Bland explained that this coefficient should be ≥0.8, while alpha equal to 0.7 is also acceptable [20]. The Cronbach’s alpha for the constructed questionnaire was 0.845, showing its high reliability.

#### Quantitative data analysis

The results of completed questionnaires with the data coding in a univariate approach was analyzed using the SPSS software package version 22.0 (SPSS Inc., Chicago, IL, USA).

## Results

### Qualitative data

A thematic framework was used to analyze the qualitative data in all the groups with 6 themes (centralized codes: F, P, C, D, E, and S) and 10 sub-themes (principal codes). Table 3 shows the definitions and indexes of six centralized codes related to the qualitative data. The centralized code of “F” involves factors relevant to the physician and divided into three sub-themes. In the principal code of F1, most of the items mentioned in the study groups included physician’s good behavior interaction in interacting with patients. The most frequent physician’s individual characteristics in the code of F2 were doctor’s neat, clean-cut appearance, gender, and etc. The influential professional parameters from the perspective of the different interviewed groups (F3) were doctor’s experience and expertise, good clinical skill, and etc. The severity of illness and need was diagnosed as the main factor in the code of P1, whereas the patient’s satisfaction in the previous examination with the physician or the patient’s trust in the physician was the key influential factor in P2. Some of the location factors in the sub-theme of C2 were geographical proximity, convenient accessibility, and cleanness of the well-equipped and stylish doctor’s office. Short waiting time, high availability to the physician in more weekdays, and the nearness of other health services to the doctor’s office or clinic were in the sub-theme of C2. The main political factor affecting the FP choice was government decisions on the distribution of physicians in different geographical areas. The code of E shows patients’ economic access to doctors such as free or low-cost visit price and private sectors. The most important social factors affecting the patients’ choice were being fellow-citizen, having a specialist degree, satisfaction and experiences of other patients. Table 4 shows the charting stage of framework from the viewpoints of HSMs and physicians.

### Quantitative data

After obtaining the data codes from the qualitative study section, a new questionnaire was designed with high validity and reliability to evaluate the quantitative data. Four hundred patients in the age range of 18–76 years (age mean of 38.6 years) participated to fill out the questionnaire. The response rate of the questionnaire was 94%. The demographic characteristics of patients are mentioned in Table 5. Most participants had a diploma and were married. Overall, the highest score among the 26 questionnaire items belonged to being a family physician specialist (4.49 ± 0.70). After that, performing careful examination with receiving a detailed medical history, and assigning enough time to visit patients were other items influencing the FP selection, respectively. However, being fellow-citizen, the patient’s tendency to same-gender doctors and doctor’s appearance were ranked with the lowest scores, respectively. The participated women and men similarly mentioned that the physician’s specialty, the complete and exact examination, as well as assigning sufficient time to visit were the most important factors influential patients’ choice. Women compared to men paid more attention to items like waiting time for a doctor’s visit, and the illness follow-up, while men were more important in the physician’s office appearance and equipment and her/his characteristics such as trust, confidentiality, and reputability (Table 6). As majority patients (81.5%) had an educational level lower than bachelor’s degree, items with the highest (having the specialist degree) and lowest (doctor’s appearance) scores were very similar to the total population of the study. In patients with a bachelor’s degree or higher (18.5%), the highest score was given the examination accuracy and receiving the correct medical history, and having the expertise of an FP, respectively. This study showed that marital status and insurance were not significant factors in choosing a physician.

**Table 5:** Demographic data of the subjects who participated in the quantitative phase

Variable	Data ^a^	
	Frequency (n)	Percentage (%)
Gender		
Man	117	29.25
Woman	283	70.75
Marriage status		
Married	319	79.75
Single	81	20.25
Educational level		
Illiterate	18	4.50
Under diploma	94	23.50
Diploma	214	53.50
Bachelor	61	15.25
Master and above	13	3.25
Health insurance status		
Yes	342	85.50
No	58	14.50

^a^The total number of participants (N_total_) was 400

**Table 6:** Ranking of the most important factors influencing FP selection based on the results of the designed questionnaire filled by the entire population

Item rank no.	Overall ranking in the total population (*n* = 400)	Mean ± SD	Ranking in the women’s population (*n* = 283)	Mean ± SD	Ranking in the men’s population (*n* = 117)	Mean ± SD
1	Being a specialist	4.49 ± 0.70	Being a specialist	4.52 ± 0.67	Being a specialist	4.41 ± 0.76
2	Careful examination with receiving a detailed medical history	4.43 ± 0.68	Careful examination with receiving a detailed medical history	4.46 ± 0.69	Careful examination with receiving a detailed medical history	4.35 ± 0.66
3	Sufficient time assigned to visit	4.28 ± 0.75	Sufficient time assigned to visit	4.32 ± 0.69	Rational prescription of drugs and medical tests	4.25 ± 0.80
4	Physicians’ intelligible expression	4.24 ± 0.76	Physicians’ intelligible expression	4.29 ± 0.73	Providing services other than visit like injection, ...	4.24 ± 0.79
5	Providing services other than visit like injection, ...	4.20 ± 0.86	Regular follow-up of patients’ illness	4.21 ± 0.77	Sufficient time assigned to visit	4.20 ± 0.86
6	Rational prescription of drugs and tests	4.18 ± 0.81	Health and illness status	4.20 ± 0.85	Physicians’ intelligible and clear expression	4.12 ± 0.80
7	Health and illness status	4.17 ± 0.84	Providing services other than visit like injection, ...	4.18 ± 0.89	Health and illness status	4.07 ± 0.81
8	Regular follow-up of patients’ illness	4.14 ± 0.83	Rational prescription of drugs and tests	4.15 ± 0.82	High trust in the doctor	4.06 ± 0.82
9	High trust in the doctor	4.10 ± 0.84	Good morals and behavior	4.14 ± 0.90	Keeping the patient’s medical secrets	4.05 ± 0.92
10	Attention to other aspects of health	4.09 ± 0.88	High trust in the doctor	4.12 ± 0.85	Attention to other aspects of health	4.03 ± 0.90
11	Keeping the patient’s medical secrets	4.07 ± 0.90	Attention to other aspects of health	4.11 ± 0.87	Regular follow-up of patients’ illness	3.98 ± 0.94
12	Good morals and behavior	4.05 ± 0.93	Keeping the patient’s medical secrets	4.07 ± 0.90	Well-equipped and stylish doctor’s office	3.89 ± 0.91
13	Proximity to the doctor’s office	4.01 ± 0.86	Proximity to the doctor’s office	4.07 ± 0.86	Proximity to the doctor’s office	3.87 ± 0.85
14	Kindness and empathy with the patient	4.00 ± 0.87	Kindness and empathy with the patient	4.06 ± 0.86	Kindness and empathy with the patient	3.87 ± 0.88
15	The receptionist’s or secretary’s behavior	3.92 ± 0.92	Waiting time to the doctor’s visit	3.95 ± 0.88	The receptionist’s or secretary’s behavior	3.82 ± 0.93
16	Well-equipped and stylish doctor’s office	3.88 ± 0.95	The receptionist’s or secretary’s behavior	3.95 ± 0.91	Satisfaction of other patients	3.82 ± 0.93
17	Waiting time to the doctor’s visit	3.88 ± 0.93	Well-equipped and stylish doctor’s office	3.88 ± 0.97	Good morals and behavior	3.82 ± 0.97
18	Satisfaction of other patients	3.81 ± 0.94	Satisfaction of other patients	3.80 ± 0.95	Doctor’s reputation	3.81 ± 1.06
19	Doctor’s reputation	3.67 ± 1.11	Doctor’s age and experience	3.64 ± 1.12	Waiting time to the doctor’s visit	3.70 ± 1.03
20	Doctor’s age and experience	3.63 ± 1.09	Doctor’s reputation	3.61 ± 1.13	Doctor’s age and experience	3.60 ± 1.03
21	Recommendation of health care workers	3.50 ± 1.01	Recommendation of health care workers	3.54 ± 0.98	Recommendation of health care workers	3.40 ± 1.03
22	More time accessibility to the doctor	3.43 ± 1.31	More time accessibility to the doctor	3.47 ± 1.27	More time accessibility to the doctor	3.32 ± 1.39
23	Suggestion by friends and acquaintances	3.25 ± 1.02	Suggestion by friends and acquaintances	3.27 ± 1.02	Physician’s appearance	3.22 ± 1.12
24	Physician’s appearance	3.24 ± 1.11	Physician’s appearance	3.25 ± 1.11	Suggestion by friends and acquaintances	3.21 ± 1.01
25	Being same gender	2.66 ± 1.34	Being same gender	2.69 ± 1.33	Being same gender	2.57 ± 1.38
26	Being a fellow-citizen	2.40 ± 1.34	Being a fellow-citizen	2.45 ± 1.33	Being a fellow-citizen	2.27 ± 1.36

*SD* standard Deviation

## Discussion

The most important factors affecting the choice of an FP by patients out of 26 items were the specialist degree in FM, followed by careful examination and history, and assigning sufficient time to visit. Bornstein conducted an exploratory cross-sectional survey of parameters influencing American patients’ choice of a primary care doctor. Results revealed that the participants highlighted relevant professional factors (e.g., the validity of FP’s degree and office appearance) and management practices (e.g., appointment times (nights and weekends)) more than the FP’s inherited characteristics (e.g., race, age, gender, etc.). Also, the most important factors found by 636 patients in choosing an FP are those that have the most significant impact on the quality of health care [21]. Mosadeghrad and Joya reported that the process and type of medicinal services provided by physicians were the most important reasons for patients’ choice in Tehran city [13]. Grol and et al. assessed the highest priority of patients to choose physicians in Europe. These factors included having adequate time during the visit, delivery of health care services under emergency conditions, the confidentiality of patients’ information, and presenting explanations required about the illness to each individual. Similarly, assigning enough time to visit patients was the main priority in choosing physicians [22]. Nouronnesa also found that the most important priorities were the physician’s skills and expertise, getting a complete medical history under the careful checkup, and how to answer a doctor to the patient’s questions [23]. In our study, receiving a detailed medical history with a careful examination was determined as the second priority after having an FP specialty. In contrast to the views of patients referring to target healthcare centers, health system managers in their interviews did not point out having a degree in FM as a priority for the effective implementation of the FP plan. This fact shows the importance of launching a specialty in FM in the country.

Results indicated that the least importance belonged to the citizenship, being the same gender, and appearance of FP. As our study area geographically is more populated by immigrants than in other cities in the country, the same dialect, ethnicity, and race from the patients’ point of view do not matter much to refer to a doctor. Similar results were reported by Bornstein in the US [21]. However, Bachmann in a qualitative study, realized that Russian-speaking migrants were less satisfied with primary care consultations compared to native Germans [24]. The cause of dissatisfaction with treatment may be attributed to the more inferior patient-physician relationship and frequent physician changes. Therefore, physicians need to be more aware of the cultural expectations of immigrants in order to better understand their needs, improve the relationship between physician and patient, and ensure equal opportunities in health care. It was earlier proved that the leading cause of patients’ choice in the UK was the proximity of physician office to patients’ home, the suggestion of patient’s friends, and previous visits of patients’ family members by the selected physician [25]. In the present study, access to the office place had relative importance, whereas the advice of a physician by the patient’s friends was of little importance. Oleszczyk in 2017 mentioned that the characteristics of patients and their physicians did not significantly affect the satisfaction and experiences of patients in Polish primary care [26]. However, our qualitative results showed that the FP’(F-code) and patients (P-code) characteristics in all interviews were influencing factors in patient satisfaction and FP selection. This finding revealed that patients’ trust in their physician plays a central role in Iran’s health system. The trust was also determined as a fundamental factor in the loyalty of French patients [27].

One of the most important factors in this study was sufficient time allocated by an FP to visit patients along with a detailed evaluation of medical history and clinical examination. This goal can be achieved if the workplace conditions and the number of referrals are well controlled. In other words, one of the essential pillars for achieving this purpose is allocating a reasonable number of referrals to each FP. On the other hand, being the same gender or fellow-citizen or having access to a physician in our research is less important than other causes. Concerning the different cultural contexts in Iran’s society, patients are willing to accept difficult access times and gender differences but referred to the physician with their preferred priority.

In the present study, there was no difference in the preferences of patients with different education levels. But Aelbrecht reported that participants with lower education were more likely to favor aspects related to emotional issues, while patients with college education paid more concerned with vocational skills issues [10]. In this study, there was a difference in the views of Iranian men and women to choose the FP. For women, the physician’s clear expression and the effective follow-up were critical, while for men, reasonable prescription of the test, and medication accompanying with medical services (such as equipped laboratory and pharmacy) was significant. This finding was affirmative to the result of Wolosin, who concluded that American women and men were more satisfied with physician-related items and the service delivery process, respectively [5]. Street in 2014 found that patients were less satisfied with primary care providers who were more likely to look at computers and be more conscientious in counseling. However, this fact was not mentioned by any of our interviewees [28].

### Study limitations and strengths

Discrimination of our study from other surveys performed in the field of FP was the design of a 26-item questionnaire using a triangular method according to the population-based data obtained from qualitative evaluations. This design method resulted in effective factors being searched from different angles, and no points were ignored in the questionnaire. On the other hand, given that the FP program and referral system were not implemented in the Tehran metropolitan area, we had to provide verbal explanations about this program and the research aims while filling out questionnaires. Recording an abstract on top of the questionnaires along with giving oral descriptions, could significantly solve this problem, although it was a time-consuming and challenging task. Due to the nascent FP program, this study was limited to the factors influencing FP selection, whereas the most important factors affecting the change or departure of the FP were not examined. Also, it was impossible to exclude desirability bias as participated doctors might have chosen answers thought to be more desirable instead of closer responses to their beliefs. The evaluation result of a broad range of factors showed that what was a top priority for patients was ignored by health managers.

## Conclusion

The present qualitative-quantitative study evaluated the patients’ preferences to select FP in Iranian primary health centers. A set of influential parameters on the FP selection was assessed after the comprehensive literature review and the face-to-face interview with physicians, patients, and HSMs based on the open-ended questions. A five-stage thematic framework analysis method was first applied to qualitatively analyze the data and then a 26-item quantitative questionnaire was used to rank the most important the influencing factor in choosing the FP according to a 5-point Likert scale. Results showed that the main factor was having a specialist degree in FM, followed by precise examinations, in-depth inquiry into the patient’s medical issues, and giving the patient enough visit time to review all outstanding health problems. Also, patients when choosing a FP paid less attention to factors such as physician’s appearance, as well as being fellow-citizen and same-gender with doctors. Accordingly, expanding this nascent medicine branch with recruiting graduates with high professional commitment in health centers may play a key role in presenting high-quality medical services with patients’ satisfaction levels.

## Data Availability

The datasets analyzed during the current study are available from the corresponding author on reasonable request.

## Abbreviations

FP: Family physician
FM: Family medicine
GP: General practitioner
SD: Specialist doctor
FMR: Family medicine resident
EPHS: Essential Package of Health Services
HSM: Health system manager
EQIs: Experienced qualitative interviewers
IEP: Internal expert panel
CVR: Content validity ratio
CVI: Content validity index
